# Risk factors in *DUX4*-positive childhood and adolescent B-cell acute lymphoblastic leukemia

**DOI:** 10.1038/s41408-024-01099-3

**Published:** 2024-07-22

**Authors:** Dagmar Schinnerl, Marion Riebler, Angela Schumich, Sabrina Haslinger, Alice Bramböck, Andrea Inthal, Marek Nykiel, Margarita Maurer-Granofszky, Oskar A. Haas, Ulrike Pötschger, Stefan Köhrer, Karin Nebral, Michael N. Dworzak, Andishe Attarbaschi, Sabine Strehl

**Affiliations:** 1 St. Anna Children’s Cancer Research Institute (CCRI), Vienna, Austria; 2 Labdia Labordiagnostik, Vienna, Austria; 3 St. Anna Children’s Hospital, Medical University of Vienna, Vienna, Austria

**Keywords:** Cancer genetics, Genetics research

## Abstract

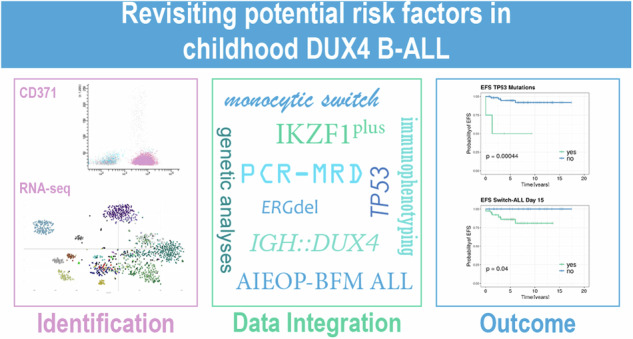

**TO THE EDITOR**:

Comprehensive integrative genomic characterization has advanced the classification of B-cell acute lymphoblastic leukemia (B-ALL) [1]. DUX4 leukemia is generally characterized by *IGH::DUX4* fusions resulting in the expression of truncated DUX4 isoforms and a distinctive gene expression signature [2–4]. Due to the cryptic nature of the rearrangement, next-generation sequencing (NGS) approaches are optimal for accurately detecting this subtype [2–4]. Other characteristic features of DUX4 leukemia include a high incidence of *ERG* deletion, CD371 cell surface expression [2–5], and a propensity to undergo a transient switch toward the monocytic lineage (swALL) early during induction therapy [6, 7].

Several studies have shown that DUX4 confers a good prognosis, however, most patients have been treated with non-standard or high-risk protocols [8–11]. In this study, we aimed to investigate the yet unknown prognostic value of DUX4 in childhood and adolescent B-ALL patients treated with AIEOP-BFM ALL regimens and to determine potential risk factors for therapy optimization.

We retrospectively investigated 1237 B-ALL patients enrolled in consecutive clinical trials conducted in Austria over more than two decades. To identify patients belonging to the DUX4 subtype, we employed an iterative combination of genetic analyses and immunophenotyping (Supplementary Fig. S1, Supplementary Methods and Notes). Sixty-six DUX4 cases were identified by RNA-seq, three additional cases by the presence of an *ERG* deletion and positive *IGH::DUX4*-specific genomic-PCR, and one genetically undefined case showed strong CD371 and CD2 expression. As this marker combination is typical of DUX4 B-ALL [5, 12] (Supplementary Table S4), we assigned the latter patient to the DUX4 group exclusively based on immunophenotyping (Supplementary Notes).

Notably, in our B-other cohort, 9.4% (3/32) of the *ERG*-deleted cases belong to other genetic groups, suggesting some caution in using *ERG* status alone to assign patients to this subtype (Supplementary Table S4). On the other hand, we confirmed the high positive/negative predictive values of CD371 expression for DUX4 B-ALL [5] (Supplementary Table S4), making immunophenotyping a fast and reliable alternative for identifying this subtype when NGS technologies are unavailable (Supplementary Notes).

In total, we classified 70 patients as DUX4 (Table 1), accounting for roughly 19% of the B-other and 6% of the entire B-ALL cohort, which is consistent with previous population-based studies [2, 4, 10, 13].

**Table 1:** Demographic and clinical characteristics of DUX4 patients treated on AIEOP-BFM ALL protocols.

Characteristics	Total (*N* = 70)	AIEOP-BFM ALL 2000 (*N* = 33)	AIEOP-BFM ALL 2009 (*N* = 24)	AIEOP-BFM ALL 2017 (*N* = 13)
*Gender, No. (%)*				
Female	33 (47.1)	15 (45.5)	10 (41.7)	8 (61.5)
Male	37 (52.9)	18 (54.5)	14 (58.3)	5 (38.5)
*Age at diagnosis, years, median, range*				
	10.3 (2.3–18.4)	9.5 (2.3–16.6)	13.2 (3.3–18.4)	9.5 (4.5–17.4)
*Initial WBC (×10*^*9*^*/L), No. (%)*				
<20	51 (72.9)	26 (78.8)	19 (79.2)	6 (46.2)
20 to <50	13 (18.6)	4 (12.1)	3 (12.5)	6 (46.2)
>50	6 (8.6)	3 (9.1)	2 (8.3)	1 (7.7)
*Prednisone response, No. (%)*				
Good	58 (82.9)	29 (87.9)	19 (79.2)	10 (76.9)
Poor	9 (12.9)	3 (9.1)	4 (16.7)	2 (15.4)
Unknown	3 (4.3)	1 (3.0)	1 (4.2)	1 (7.7)
*PCR-MRD risk group, No. (%)*				
LR	6 (8.6)	5 (15.2)	1 (4.2)	0
IR	47 (67.1)	26 (78.8)	12 (50.0)	9 (69.2)
HR	15 (21.4)	1 (3.0)	10 (41.7)	4 (30.8)
Unknown	2 (2.9)	1 (3.0)	1 (4.2)	0
*FCM-MRD risk group, No. (%)*				
FLR	17 (24.3)	9 (37.5)^a^	5 (20.8)	3 (23.1)
FMR	42 (60.0)	21 (87.5)^a^	13 (54.2)	8 (61.5)
FHR	11 (15.7)	3 (12.5)^a^	6 (25.0)	2 (15.4)
*Study risk group, No. (%)*				
SRG	6 (8.6)	5 (15.2)	1 (4.2)	0
MRG	43 (61.4)	24 (72.7)	11 (45.8)	8 (61.5)
HRG	21 (30.0)	4 (12.1)	12 (50.0)	5 (38.5)

^a^FCM-MRD was assessed but not implemented as risk stratifying parameter in the AIEOP-BFM ALL 2000 study.

*LR* low risk, *IR* intermediate risk, *HR* high-risk, *FLR* flow cytometry low risk, *FMR* flow cytometry medium risk, *FHR* flow cytometry high-risk, *SRG* standard risk group, *MRG* medium risk group, *HRG* high-risk group, *FCM* flow cytometry, *WBC* white blood cell count.

Most of the 70 DUX4 patients were treated according to the medium risk (MRG) or high-risk (HRG) protocols of the respective clinical trials (Table 1, Supplementary Table S2). Only 7.1% (5/70) of patients relapsed, of which 80% (4/5) died, 60% (3/5) of progressive disease, indicating a need for improved salvage therapy. One additional patient died early while still on therapy, bringing the total number of deaths to 7.1% (5/70). At a median follow-up of 7.4 years (range 0.7–17.4) the estimated 5-year and 10-year event-free survival (EFS) of all patients was 92.0 ± 3.5% and 89.6 ± 4.1%, while the 5-year and 10-year overall survival (OS) was 95.1 ± 2.8% and 88.1 ± 5.7%, and the 5-year and 10-year cumulative incidence of leukemia-related events (CIL) was 8.0 ± 3.5% and 10.4 ± 4.2%, respectively (Fig. 1A). Consistent with previously reported survival rates [8–11], patients with DUX4 leukemia treated according to AIEOP-BFM ALL protocols also have a very good overall outcome.

**Fig. 1:**
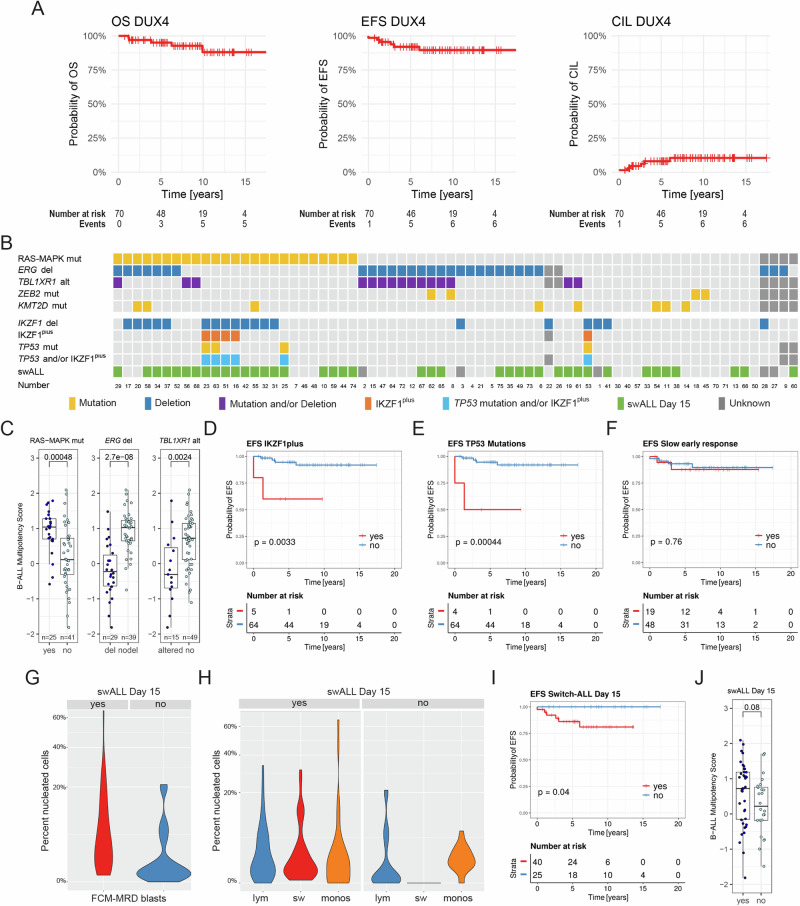
Genetic features, developmental states, swALL, and outcome of DUX4 patients. **A** Kaplan–Meier survival curves of event-free (EFS) and overall survival (OS) as well as cumulative incidence of leukemia-related events (CIL) of all DUX4 cases (*n* = 70). **B** Summary of secondary genetic alterations found in DUX4 cases. mut mutation, del deletion, alt mutation and/or deletion, IKZF1^plus^ deletion profile, swALL DUX4 ALL with monocytic lineage switch determined by flow cytometry on day 15 of treatment; depicted numbers correspond to patient numbers in Supplementary Table S2. **C** B-ALL multipotency score of DUX4 samples with different alterations. mut mutation, del deletion, alt mutation, and/or deletion; numbers of cases per subgroup are depicted at the bottom of the boxplots; Wilcoxon rank sum *p*-value. **D**–**F** Kaplan–Meier survival curves of EFS based on (**D**) IKZF1^plus^ deletion profile; red, positive, blue, negative. **E** *TP53* mutation status; red, *TP53*-mutated, blue, *TP53* wild-type. **F** slow early response (SER); red, yes, blue, no. Log-ranks test *p*-values for EFS. **G** Violin plot showing the distribution of FCM-MRD blasts (combining lymphoblasts [lym] and switch blasts [sw]) in DUX4 swALL and non-swALL. **H** Violin plots showing the distribution of lym, sw-blasts, and monocytes (monos) in DUX4 swALL and non-swALL. **I** Kaplan–Meier survival curves of EFS based on DUX4 ALL with monocytic lineage switch (swALL); red, yes, blue, no. Log-ranks test *p*-values for EFS. **J** Boxplots showing B-ALL multipotency score for swALL vs. non-swALL DUX4 cases; Wilcoxon rank sum *p*-value.

Recently, it has been demonstrated that in B-ALL, distinct developmental states are associated with genomic alterations and clinical characteristics [14]. Therefore, we analyzed the frequency of recurrent secondary genetic alterations and their association with developmental stage and impact on outcome (Fig. 1B, Supplementary Fig. S2A, Supplementary Table S5). While, as previously shown [14], samples with RAS-MAPK-pathway mutations (37.9%, 25/66) were associated with the early lymphoid state and higher B-ALL multipotency scores, those with *ERG* deletion (43%, 29/68) and/or *TBL1XR1* alterations (23.4%, 15/64), had significantly lower B-ALL multipotency scores (Fig. 1C), however, we did not observe any effect on outcome (Supplementary Fig. S2C–E). Neither *KMT2D* (12.1%, 8/66) nor *ZEB2* (6.1%, 4/66) mutation was associated with any developmental state or had any impact on survival (Supplementary Fig. S2B, F, G).

Samples with *IKZF1* deletion and the IKZF1^plus^ deletion profile [15] showed higher B-ALL multipotency scores (Supplementary Fig. S2B). As previously reported by others [4, 9, 12], *IKZF1* deletion alone (27.1%, 19/70) had no impact on outcome (Supplementary Fig. S2H), while IKZF1^plus^ patients (7.2%, 5/69) had a significantly worse 5-year EFS of 60 ± 21.9% vs. 94.5 ± 3.1% (*p* = 0.0033) (Fig. 1D).

Notably, the IKZF1^plus^ deletion profile coincided with *TP53* mutation (60%, 3/5; Fig. 1B, Supplementary Table S6). At diagnosis, we detected *TP53* mutations in 5.7% (4/70) of patients co-occurring with deletions of the second allele, and in one patient, who died shortly after relapse, a *TP53* mutation was present only at this time-point. Although the number of *TP53*-mutated DUX4 patients in our cohort is low, they had a significantly worse outcome (5-year EFS 50 ± 25% vs. 94.5 ± 3.1%, *p* = <0.001; Fig. 1E), confirming their reported dismal survival [11].

The risk stratification parameters changed from the AIEOP-BFM ALL 2000 to the 2009/2017 trials. Therefore, in addition to using the actual risk groups (RGs) for outcome analysis, we performed a virtual RG classification applying the AIEOP-BFM ALL 2017 risk factors (Supplementary Fig. S3A, Supplementary Table S7).

We found no significant difference in the survival probabilities of DUX4 patients stratified into the actual RGs according to the respective protocol parameters (Supplementary Fig. S3B, Supplementary Table S5). Remarkably, while the actual PCR-based measurable residual disease (PCR-MRD) RGs showed significantly different outcomes (5-year EFS LR 100 ± 0%, IR 97.4 ± 2.5%, HR 69.6 ± 12.9%, *p* = 0.0061), virtual re-stratification diminished the predictive value of PCR-MRD (5-year EFS LR 100 ± 0%, IR 96.7 ± 3.3%, HR 81.8 ± 8.3%, *p* = 0.2; Supplementary Fig. S3C, D, Supplementary Table S5).

The changes in MRD-RG classification are based on a slow early response (SER) to therapy, defined as PCR-MRD of ≥5 × 10^−4^ after induction therapy and any PCR-MRD positivity <5 × 10^−4^ after consolidation therapy, and used as high-risk stratification factor only in the AIEOP-BFM ALL 2009/2017 clinical trials [15]. Remarkably, in our DUX4 cohort, SER (28.4%, 19/67 with available data) to treatment had no significant impact on survival (5-year EFS 87.7 ± 8.2% vs. 93.1 ± 3.8%, *p* = 0.76; Fig. 1F). This is of particular interest, because 33.3% (8/24) of the DUX4 MRG patients treated according to the AIEOP-BFM ALL 2000 regimen, would nowadays be assigned to the HRG (*n* = 6 SER, *n* = 2 SER plus FCM-MRD ≥ 10% at day 15), however, none of these patients relapsed or died despite non-HR treatment (median follow-up 9.6 years, range 2.1–15.4 years; Supplementary Fig. S3A). This finding suggests that in the absence of other high-risk parameters, DUX4 SER patients may not require HR treatment.

Due to the occurrence of the swALL phenomenon (changes in lymphoid antigen expression and scatter levels representing gradual lympho-monocytoid transdifferentiation, accumulation of mature-appearing monocytes) [7], flow cytometry (FCM) based MRD monitoring may be challenging. Applying the current recommendation to include lymphoblasts and switch blasts for FCM-MRD assessment [6], on day 15 of treatment patients with swALL showed a significantly higher MRD burden than those with non-swALL (median 3.9%, range 0.1–65% vs. median 0.21%, range 0.0–21%; Fisher *p*-value < 0.001; Fig. 1G) and those with swALL also had higher lymphoblast counts than non-swALL cases (Fig. 1H).

All patients without detectable swALL by FCM on day 15 (38.5%, 25/65 with available data) remained in long-term remission, while those with swALL (61.5%, 40/65) had a worse outcome (5-year EFS 100% vs. 86.1 ± 5.8%, *p* = 0.04; Fig. 1I). Unexpectedly, DUX4 swALL did not show higher abundances of earlier developmental stages or a higher B-ALL multipotency score than non-swALL (Fig. 1J). We have no reasonable explanation for this finding, however, developmental and phenotypic plasticity assessed by transcriptome profiling and immunophenotyping, respectively, may reflect different characteristics of the blast cells.

Although not reaching statistical significance, DUX4 patients with <0.1% FCM-MRD lymphoblasts at day 15 (27.7%, 18/65 with available data) did not experience an event (Supplementary Fig. S4A) (83.3%, 15/18 non-HR treatment; Supplementary Table S8). A similar trend was also observed within the group of DUX4 patients with swALL (Supplementary Fig. S4B). Applying the currently used ≥10% cutoff for FCM-MRD assessment of high-risk disease, including only lymphoblasts better discriminated patients with a worse outcome than including both lymphoblasts and switch blasts (Supplementary Fig. S4C, D) or adding the monocyte-like cells (Supplementary Fig. S4E).

Notably, the best discrimination of outcome was achieved using FCM-based MRD measurement at day 15, including both lymphoblasts and switch blasts and a cutoff of 1%, as used in US-based protocols [8] (5-year EFS 100% vs. 83.4 ± 6.8%, *p* = 0.015; Supplementary Fig. S5A). Accordingly, though also not a risk stratification criterion in AIEOP-BFM ALL studies, PCR-MRD < 10^−2^ vs. ≥10^−2^ measured on day 15 also clearly distinguished patients with long-term EFS from those who experienced an event (Supplementary Fig. S5B).

Collectively, we show that patients with DUX4 B-ALL generally have a favorable outcome when treated according to AIEOP-BFM ALL protocols. However, recurrent secondary genetic alterations, such as the IKZF1^plus^ deletion profile and *TP53* mutation or a switch to the monocytic lineage during treatment, are indicative of a worse prognosis. The relevance of MRD response kinetics, particularly slow early response to therapy, the significance thresholds, and cell populations considered for FCM-MRD assessment may differ between DUX4 B-ALL and other subtypes. Therefore, we advocate a roadmap to revisit the prognostic factors in DUX4 patients across clinical trials to ultimately refine risk stratification and optimize therapy for this subgroup of leukemia.

### Supplementary information

Supplementary File

Supplementary Table S1

Supplementary Tables S2 & S3

## Data Availability

The htseq count table of the DUX4 cases is included in Supplementary Table S1. Due to patient privacy, all relevant raw data of this study are only available from the corresponding author upon reasonable request.

## References

[1] Pagliaro L, Chen S-J, Herranz D, Mecucci C, Harrison CJ, Mullighan CG, et al. Acute lymphoblastic leukaemia. Nat Rev Disease Prim. 2024;10:41. 38871740 10.1038/s41572-024-00525-x

[2] Lilljebjorn H, Henningsson R, Hyrenius-Wittsten A, Olsson L, Orsmark-Pietras C, von Palffy S, et al. Identification of ETV6-RUNX1-like and DUX4-rearranged subtypes in paediatric B-cell precursor acute lymphoblastic leukaemia. Nat Commun. 2016;7:11790. 27265895 10.1038/ncomms11790 PMC4897744

[3] Yasuda T, Tsuzuki S, Kawazu M, Hayakawa F, Kojima S, Ueno T, et al. Recurrent DUX4 fusions in B cell acute lymphoblastic leukemia of adolescents and young adults. Nat Genet. 2016;48:569–74. 27019113 10.1038/ng.3535

[4] Zhang J, McCastlain K, Yoshihara H, Xu B, Chang Y, Churchman ML, et al. Deregulation of DUX4 and ERG in acute lymphoblastic leukemia. Nat Genet. 2016;48:1481–9. 27776115 10.1038/ng.3691 PMC5144107

[5] Schinnerl D, Mejstrikova E, Schumich A, Zaliova M, Fortschegger K, Nebral K, et al. CD371 cell surface expression: a unique feature of DUX4-rearranged acute lymphoblastic leukemia. Haematologica. 2019;104:e352–5. 30705095 10.3324/haematol.2018.214353 PMC6669149

[6] Buldini B, Varotto E, Maurer-Granofszky M, Gaipa G, Schumich A, Bruggemann M, et al. CD371+ pediatric B-cell acute lymphoblastic leukemia: propensity to lineage switch and slow early response to treatment. Blood. 2024;143:1738–51. 38215390 10.1182/blood.2023021952

[7] Novakova M, Zaliova M, Fiser K, Vakrmanova B, Slamova L, Musilova A, et al. DUX4r, ZNF384r and PAX5-P80R mutated B-cell precursor acute lymphoblastic leukemia frequently undergo monocytic switch. Haematologica. 2021;106:2066–75. 32646889 10.3324/haematol.2020.250423 PMC8327733

[8] Jeha S, Choi J, Roberts KG, Pei D, Coustan-Smith E, Inaba H, et al. Clinical significance of novel subtypes of acute lymphoblastic leukemia in the context of minimal residual disease-directed therapy. Blood Cancer Discov. 2021;2:326–37. 34250504 10.1158/2643-3230.BCD-20-0229 PMC8265990

[9] Li Z, Lee SHR, Chin WHN, Lu Y, Jiang N, Lim EH, et al. Distinct clinical characteristics of DUX4- and PAX5-altered childhood B-lymphoblastic leukemia. Blood Adv. 2021;5:5226–38. 34547766 10.1182/bloodadvances.2021004895 PMC9152998

[10] Schwab C, Cranston RE, Ryan SL, Butler E, Winterman E, Hawking Z, et al. Integrative genomic analysis of childhood acute lymphoblastic leukaemia lacking a genetic biomarker in the UKALL2003 clinical trial. Leukemia. 2023;37:529–38. 36550215 10.1038/s41375-022-01799-4 PMC9991913

[11] Ueno H, Yoshida K, Shiozawa Y, Nannya Y, Iijima-Yamashita Y, Kiyokawa N, et al. Landscape of driver mutations and their clinical impacts in pediatric B-cell precursor acute lymphoblastic leukemia. Blood Adv. 2020;4:5165–73. 33095873 10.1182/bloodadvances.2019001307 PMC7594377

[12] Clappier E, Auclerc MF, Rapion J, Bakkus M, Caye A, Khemiri A, et al. An intragenic ERG deletion is a marker of an oncogenic subtype of B-cell precursor acute lymphoblastic leukemia with a favorable outcome despite frequent IKZF1 deletions. Leukemia. 2014;28:70–7. 24064621 10.1038/leu.2013.277

[13] Krali O, Marincevic-Zuniga Y, Arvidsson G, Enblad AP, Lundmark A, Sayyab S, et al. Multimodal classification of molecular subtypes in pediatric acute lymphoblastic leukemia. NPJ Precis Oncol. 2023;7:131. 38066241 10.1038/s41698-023-00479-5 PMC10709574

[14] Iacobucci I, Zeng AGX, Gao Q, Garcia-Prat L, Baviskar P, Shah S, et al. Single cell dissection of developmental origins and transcriptional heterogeneity in B-cell acute lymphoblastic leukemia. 2023. 10.1101/2023.12.04.569954.

[15] Stanulla M, Dagdan E, Zaliova M, Moricke A, Palmi C, Cazzaniga G, et al. IKZF1(plus) defines a new minimal residual disease-dependent very-poor prognostic profile in pediatric B-cell precursor acute lymphoblastic leukemia. J Clin Oncol. 2018;36:1240–9. 29498923 10.1200/JCO.2017.74.3617

