# Supplementary material for: Risk factors in DUX4-positive childhood and adolescent B-cell acute lymphoblastic leukemia
Source: Blood Cancer J. 2024 Jul 22;14(1):119. doi: 10.1038/s41408-024-01099-3 (PMC11263571; doi:10.1038/s41408-024-01099-3)
Supplement: Supplementary file 1 — Supplementary File [file 41408_2024_1099_MOESM1_ESM.docx]

Supplementary Information

Risk factors in *DUX4*-positive childhood and adolescent B-cell acute lymphoblastic leukemia

Schinnerl *et al.*

Contents

[1 Supplementary Material and Methods 3](#_Toc169696766)

[1.1 Whole transcriptome sequencing (RNA-seq) 4](#_Toc169696767)

[1.2 Bioinformatics analysis 5](#_Toc169696768)

[1.2.1 Bioinformatic approach for the identification of DUX4 cases 5](#_Toc169696769)

[1.2.2 Predicting of developmental state abundance 6](#_Toc169696770)

[1.2.3 Detection of hot spot mutations 6](#_Toc169696771)

[1.3 Immunophenotyping 7](#_Toc169696772)

[1.4 Assessment of measurable residual disease (MRD) 7](#_Toc169696773)

[1.5 Risk stratification according to AIEOP-BFM ALL 2017 criteria 8](#_Toc169696774)

[1.6 Statistical analysis 8](#_Toc169696775)

[2 Supplementary Notes 10](#_Toc169696776)

[Identification of DUX4 cases 10](#_Toc169696777)

[3 Supplementary Tables 12](#_Toc169696778)

[Supplementary Table S4 12](#_Toc169696779)

[Supplementary Table S5 13](#_Toc169696780)

[Supplementary Table S6 14](#_Toc169696781)

[Supplementary Table S7 15](#_Toc169696782)

[Supplementary Table S8 16](#_Toc169696783)

[4 Supplementary Figures 17](#_Toc169696784)

[Supplementary Figure S1 17](#_Toc169696785)

[Supplementary Figure S2 18](#_Toc169696786)

[Supplementary Figure S3 20](#_Toc169696787)

[Supplementary Figure S4 21](#_Toc169696788)

[Supplementary Figure S5 22](#_Toc169696789)

[5 Supplementary Files 23](#_Toc169696790)

[Supplementary Table S1 23](#_Toc169696791)

[Supplementary Table S2 23](#_Toc169696792)

[Supplementary Table S3 23](#_Toc169696793)

[6 Supplementary References 24](#_Toc169696794)

# Supplementary Material and Methods

**Patient cohort**

Between 06/1999-06/2023 1237 B-ALL patients including 52 with Down Syndrome-associated ALL (DS-ALL) were registered in the Austrian AIEOP-BFM ALL 2000 (NCT00430118), AIEOP-BFM ALL 2009 (NCT01117441), and AIEOP-BFM ALL 2017 (NCT03643276) as well as EsPhALL (NCT00287105, NCT01460160, NCT03007147) clinical trials. In accordance with the Declaration of Helsinki, patients were included in the respective study after obtaining informed consent from the patients, their parents, or legal guardians. The project was approved by the Ethics Committee of the Medical University of Vienna (ethical vote 2190/2018) and the use of surplus human material authorized by the institutional review board.

**Conventional genetic analysis**

Genetic analysis for routine diagnostics included testing for *ETV6::RUNX1*, *TCF3::PBX1*, *BCR::ABL1*, *TCF3::HLF*, *KMT2A::AFF1*, other *KMT2A* rearrangements, high hyperdiploidy, near haploidy, low hypodiploidy, and iAMP21, and was mainly performed using fluorescence in situ hybridization (FISH) and RT-PCR assays, karyotyping, and DNA-index according to standard protocols. Over time, the latter two were replaced by single nucleotide polymorphism (SNP) array analysis, which was also employed for the retrospective analysis of B‑other cases. Due to lack of material, nine (0.7%) of the 1237 cases could not be fully analyzed and their main genetic subtype not determined. The 29.6% of cases (364/1228; including 36 DS‑ALL patients) lacking the well-established genetic alterations were classified as B-other and subjected to further analysis (Supplementary Fig. S1).

FISH screening for *ABL1*, *ABL2*, *PDGFRB/CSF1R*, *JAK2*, *ZNF384*, *MEF2D*, *ETV6*, and *IGH* rearrangements was conducted with commercially available probes. Apart from the detection of copy number alterations (CNAs), SNP array data were also evaluated for unbalanced rearrangements indicating the presence of a fusion gene [1,2]. If either FISH and/or SNP array data indicated the presence of a fusion gene, it was verified by targeted RT‑PCR when feasible.

CytoScan HD arrays were analyzed with the Chromosome Analysis Suite version 3.0-4.3 (Applied Biosystems, Thermo Fisher Scientific, Waltham, MA, USA) as previously described [3]. When SNP array analysis was not possible, data from multiplex ligation-dependent probe amplification (MLPA) using the SALSA MLPA P335 ALL-IKZF1 kit (MRC Holland, Amsterdam, The Netherlands) were used for CNA detection of the targeted genes as described [4].

The IKZF1^plus^ deletion profile was defined as *IKZF1* deletion co-occurring with at least one other deletion of either *CDKN2A* (homozygous or heterozygous), *CDKN2B* (homozygous), *PAX5*, or of the PAR1 region resulting in the *P2RY8::CRLF2* fusion in the absence of *ERG* deletion [5]. Clearly detectable but only subclonal deletion patterns were also classified as IKZF1^plus^ positive.

## Whole transcriptome sequencing (RNA-seq)

All cases which were assigned to one of the sentinel genetic subtypes (i.e. high hyperdiploid, hypodiploid, *ETV6::RUNX1*, *TCF3::PBX1*, *BCR::ABL1*, *KMT2A::AFF1*, *KMT2A*r, *TCF3::HLF*, iAMP21) during routine diagnostic work-up, were in principle excluded from further genetic analysis. However, some selected cases from these subtypes were also subjected to RNA-seq for bioinformatics analysis purposes including hierarchical clustering and gene expression profiling as described in more detail further below.

RNA-seq was conducted as previously described [6]. In brief, high quality total RNA was isolated from bone marrow aspirates or peripheral blood containing >70% blast cells using the QIAamp RNA Blood Mini Kit (Qiagen, Hilden, Germany) or TRIZOL reagent (Invitrogen; Thermo Fischer Scientific). Library preparation and RNA-seq were outsourced to the Next Generation Sequencing (NGS) Facility at the Vienna BioCenter Core Facilities (VBCF; https://www.viennabiocenter.org/ vbcf/next-generation-sequencing/). Stranded libraries were prepared from 200-500 ng total RNA (RNA integrity number; RIN value >7) with the NEB Next Ultra II Kit including poly(A)+ enrichment using oligo(dT)-beads. Paired‑end (2×125 bp or 2×150 bp) RNA-seq with an average read depth of approximately 40 million reads per sample was performed on an Illumina HiSeq2500 or NovaSeq instrument.

## Bioinformatics analysis

Reads were aligned against human genome GRCh38 without alt loci using STAR-2.6.0c1 [7]. Read counts were generated using HTSeq (version 0.11.0) in union mode, ignoring secondary and supplementary alignments using genecode annotation v31. Htseq raw count tables of DUX4 samples can be found in Supplementary Table S1. Further analysis was performed in R (version 4.3.2) statistical environment and visualizations were generated using ggplot2 (version 3.4.1) [8].

### Bioinformatic approach for the identification of DUX4 cases

DUX4 cases were mainly identified by RNA-seq using a combination of different bioinformatics strategies.

Fusion gene detection was performed using FusionCatcher (version 1.00) [9], Arriba (version 2.1.0) [10], and CICERO (version 0.3.0) [11].

As not in all DUX4 cases fusion callers reliably detect a rearrangement, in addition supervised hierarchical clustering and gene expression classification were conducted. For this purpose, the count tables generated from RNA-seq data of B-ALL samples with various genetic subtypes included in the Real-time Clinical Genomics (RTCG) [12], the Genomes 4 Kids (G4K) [12,13], the Pediatric Cancer Genome Project (PCGP) , and the Pan-Acute Lymphoblastic Leukemia (PanALL) studies [14-21] were downloaded from the St. Jude Cloud (https://www.stjude.cloud/) [22]. Count tables were imported into R using the corresponding function from DESeq2 [23] and variance transformation was performed. Blacklist genes, including, for example, hemoglobin genes, were excluded from further analysis. Limma’s removeBatchEffect function was used to remove unwanted bias due to differences in library preparation and sequencing (covariates library strandedness, selection protocol, read length) and using “B‑ALL subtype” in the design matrix to preserve biological differences.

As DUX4 B-ALL shows a highly distinct gene expression profile, published gene sets were used to perform supervised hierarchical clustering [19,24,25] using the pheatmap function.

For gene expression classification, dimension reduction Uniform Manifold Approximation and Projection (UMAP) was performed using the uwot package. For this purpose, the top 1000 genes with the highest median absolute deviation, n_neighbors=40 and min_dist =0.5 were used. In addition, gene expression classifiers (ALLCatchR [26], ALLSorts [27]) trained on B‑ALL data were employed.

When RNA-seq was not feasible due to the unavailability of suitable material, CD371 expression was assessed by flow cytometry (FCM) and *ERG* deletion by SNP array analysis. In suspicious cases, but not as primary screening approach, an *IGH::DUX4*-specific genomic PCR was performed (Lilljebjörn *et al*., manuscript in preparation).

### **Predicting** of developmental state abundance

The prediction of the B-ALL development state abundance and the B-ALL multipotency score were performed as described [28]. In brief, downloaded count data from the St. Jude Cloud and the count data from the DUX4 samples of this study were combined and vst-transformed. Normalized count data were multiplied with the model weights reported by Iacobucci *et al.* [28]. Results were standardized for each development stage and different groups were compared using Wilcoxon rank sum test.

### Detection of hot spot mutations

For mutation calling in selected hot spot regions a workflow following the Genome Analysis Toolkit (GATK) Best Practices recommendations and Ensembl Variant Effect Predictor (VEP) annotation was employed [29-33]. The analyzed genes included key genes involved in hematopoiesis and/or lymphoid development, RAS-MAPK signaling, JAK-STAT signaling, epigenetic regulation, glucocorticoid receptor signaling, and *TP53*.

In addition, samples without available RNA-seq data were screened for *TP53* mutations. Genomic PCR assays amplifying exons 4-5, exon 6, exons 7-9, and exon 10 were performed according to standard procedures using HotStart Polymerase (Qiagen). Amplification products were directly sequenced, and sequence analysis was conducted using the CLC Main Workbench 22.0.2 (Qiagen).

## Immunophenotyping

Immunophenotyping by FCM with a standardized marker panel was conducted for diagnostic purposes and the classification of antigen expression into strong/weak/negative according to the AIEOP‑BFM consensus guidelines [34,35]. As patients were recruited over more than two decades, antigen panels have been progressively optimized, resulting in variations in the markers studied in each patient. For retrospective sample analysis, the current standardized panel, including CD371, CD2, and CD56 was used as described elsewhere [36].

## Assessment of measurable residual disease (MRD)

FCM-based bone marrow MRD levels on day 15 of therapy were assessed using standardized methods [34,35,37,38]. Antibody panels have evolved over time to include antibodies and antibody combinations proven of highest performance for MRD detection, initially using 4-color [39], and later on 8- or 9-color panels [37]. Since 2017, a dried format tube (DuraClone, Beckman Coulter) including CD45Kro, CD58FITC, CD34ECD, CD10PC5.5, CD19PC7, CD38AA700, CD20AA750 in conjunction with liquid patient-specific “drop-in” antibodies (e.g. CD371PE, clone 50C1; BD Biosciences or BioLegend) as well as Syto41 for gating nucleated cells was used for MRD detection and determination of monocytic switch [37,40]. Blasts with decreased expression of CD19, elevated side scatter (SSC) and/or strong positivity with CD34/CD58 were classified as “switch blasts” (swBlasts). “Lymphoblasts” were characterized by stronger CD19 expression, usually lower SSC and immunophenotypic characteristics resembling those at diagnosis. To improve the comparability of FCM-MRD results, samples were re‑gated according to current guidelines and re-classified into low (<0.1%), medium (≥0.1%, <10%) and high (≥10%) FCM-MRD risk groups according to (i) lymphoblasts, (ii) lymphoblasts plus switch blasts, and (ii) lymphoblasts plus switch blasts plus monocytes (CD371 strong positive, CD19 negative, SSC elevated), respectively.

Bone marrow MRD assessment by real-time quantitative PCR (PCR-MRD) of immunoglobulin and T-cell receptor (Ig/TCR) rearrangements was performed on day 15, TP1 (day 33; after induction therapy), and TP2 (day 78 or day 96; after consolidation therapy) of treatment according to standardized procedures [41,42]. Slow early response (SER) to therapy was defined as PCR‑MRD at TP1 ≥5x10^-4^ and any PCR-MRD positivity below 5x10^-4^ at TP2 [43].

## Risk stratification according to AIEOP-BFM ALL 2017 criteria

As the risk-stratifying criteria used in the three consecutive AIEOP-BFM ALL trials changed over time, a virtual risk stratification applying the parameters used in the AIEOP-BFM ALL 2017 clinical study was performed. The criteria resulting in a change of risk stratification were: (i) SER is considered MRD high-risk (HR) and patients are enrolled in the HR arm, (ii) patients with FCM-MRD ≥10% blasts on day 15 are also treated in the HR arm, while (iii) poor prednisone response on day 8 of therapy (>1.000 blasts per µl blood) alone is no longer considered as HR criterion.

## Statistical analysis

Survival analysis was performed in R (version 4.3.2) statistical environment employing ggsurvfit (version 1.0.0) and tidycmprsk (version 1.0.0) functions. Overall survival (OS) and event-free survival (EFS) rates were calculated using the Kaplan-Meier method [44] and compared by the log-rank test. The cumulative incidence of leukemia-related events (CIL) was calculated using the method of Kalbfleisch and Prentice [45] and compared with the Gray test.

OS and EFS/CIL were defined as the time from diagnosis to the date of last follow-up or death and to the date of last follow-up or first adverse event, respectively. In case of remission failure, the EFS/CIL-time to event was set to zero. Adverse events were defined as the failure to achieve remission (early death, refractory disease), relapse at any site, death during complete remission (CR), the development of a second malignancy or death of any cause.

Those parameters reaching statistical significance (*TP53* mutation, IKZF1^plus^ genotype, and switch ALL, FCM-based MRD measurement at day 15 including both lymphoblasts and switch blasts and a cutoff of 1%) in the univariate analysis were further subjected to multivariate EFS analysis using a Cox proportional hazards regression model [46]. In case of monotone likelihood, Firth’s penalized maximum likelihood reduction method was employed using the coxphf package (version 1.13.1). In addition to the parameters of interest, age (continuous variable), white blood cell count (continuous variable), gender, and the actual PCR-MRD risk group categorized as high-risk (HR) vs non-HR were used as covariates. None of the tested parameters was significant (p<0.05) in the multivariate analysis.

Other comparisons were performed using Fisher’s exact or Wilcoxon rank sum tests. For all tests, p-values <0.05 were considered statistically significant.

# Supplementary Notes

## Identification of DUX4 cases

Out of 364 (29.4%, 364/1237) cases belonging to the B-other subtype, 244 were subjected to RNA‑seq at diagnosis, relapse, or both, of which 66 showed a gene expression signature typical of DUX4 leukemia [19,24,25]. In 62 of these cases, we detected an *IGH::DUX4* and in one an *IGH::DUX4* and a concomitant *ZNF384::DUX4* fusion. In the remaining three cases we did not find any evidence for a *DUX4* rearrangement, but the gene expression profile and the employed classifiers clearly assigned them to this subtype of leukemia (Supplementary Tables S2 and S3).

Given the significant associated of DUX4 leukemia with *ERG* deletion, it is considered a surrogate marker. SNP array data were available of 325 (89.3%, 325/364) of all B-other cases and identified 9.8% (32/325) with *ERG* deletion, 26 belonging to the 66 DUX4 cases detected by RNA-seq. In three *ERG*-deleted cases without RNA-seq data we verified the presence of an *IGH::DUX4* rearrangement using a genomic PCR assay (Lilljebjörn *et al*., manuscript in preparation), bringing the number of DUX4 patients to 69.

Notably, the remaining three *ERG*-deleted cases lacked the DUX4-specific gene expression profile as determined by RNA-seq (in two cases conducted at diagnosis and in one only at relapse). Curation of the SNP array data revealed the presence of *ERG* deletions of exons 3-7 in two cases and only of exons 1 or 1-3, depending on the isoform (NM_182918 ERG ex1; NM_004449 ERG ex1-3) in the third case. Thus, 9.4% (3/32) of the *ERG*-deleted cases detected in our B-other cohort belong to other genetic groups.

In the remaining 117 cases (Supplementary Fig. S1), we conducted comprehensive FISH, targeted RT-PCR, and SNP array analysis as well as in selected cases mutation screening for PAX5 P80R and IKZF1 N159Y mutations. As virtually all subtype-defining genetic alterations are mutually exclusive, we considered 88 cases with any specific gene rearrangement or mutation as non-DUX4.

Although *IGH::DUX4* rearrangements are usually cryptic and generated by the insertion of 1‑2 D4Z4 repeats containing the *DUX4* gene into the *IGH* locus [19,47,48], by FISH analysis using *IGH* specific dual-color break-apart probes we detected a split signal pattern in 20.0% (14/70) of cases in ≥ 20% of interphase nuclei, indicating a more complex nature of the rearrangement.

As a second approach, we used immunophenotyping to identify DUX4 cases. First, we confirmed the significant correlation between CD371 surface maker expression and DUX4 [6]: 96.49% (55/57 with available FCM data) of DUX4 samples were positive for CD371 (49 strong, 6 weak), while only 3.5% (2/57) were negative, confirming the very low false-negative rate (Supplementary Table S4).

Of 206 B-other cases analyzed by FCM for both CD371 and CD2 expression, the latter associated with *ERG* deletion [11,14], all double-positive samples (n=37) were DUX4. In addition, one of the remaining 29 genetically undefined B-other cases, showed strong expression of CD371 and CD2, and we therefore assigned this patient, solely based on its immunophenotype to the DUX4 group (Supplementary Table S4).

Notably, two of the three *ERG*-deleted cases not showing the gene expression profile typical of DUX4 and investigated by FCM were also CD371 and CD2 negative.

In addition, we observed that of the 60 DUX4 cases analyzed for CD34 expression and classified according to the BFM guidelines [36,37], 98.3% (59/60) showed a strong and only 1.7% (1/60) a weak expression of CD34, but the latter case was CD371 strong. Furthermore, CD56/CD2 double-positivity seen in 21.7% (13/60) of cases was exclusive to DUX4, suggesting that this antigen combination is highly specific for this genetic subtype, albeit with a relatively low sensitivity.

Together, since only 4.9% (18/364) of our B-other cohort had insufficient genetic and/or immunologic data to exclude or include them in the DUX4 group, we are confident that we have identified virtually all cases belonging to the DUX4 subtype.

# Supplementary Tables


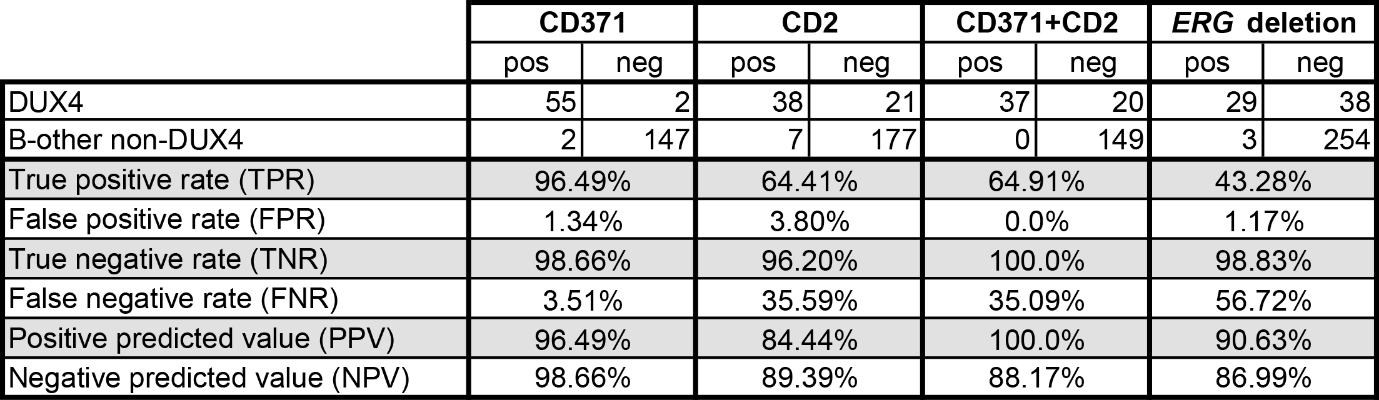


Supplementary Table S4**.** **Error rates of potential surrogate markers.** The numbers of DUX4 and non-DUX4 B-other patients positive (strong or weak) or negative for CD371, or CD2, or CD371 and CD2 surface marker expression, as determined by flow cytometry are summarized. Error rates and predictive values of the surrogate markers are provided. The patient classified as DUX4 based on immunophenotype alone was excluded for error rate calculations.


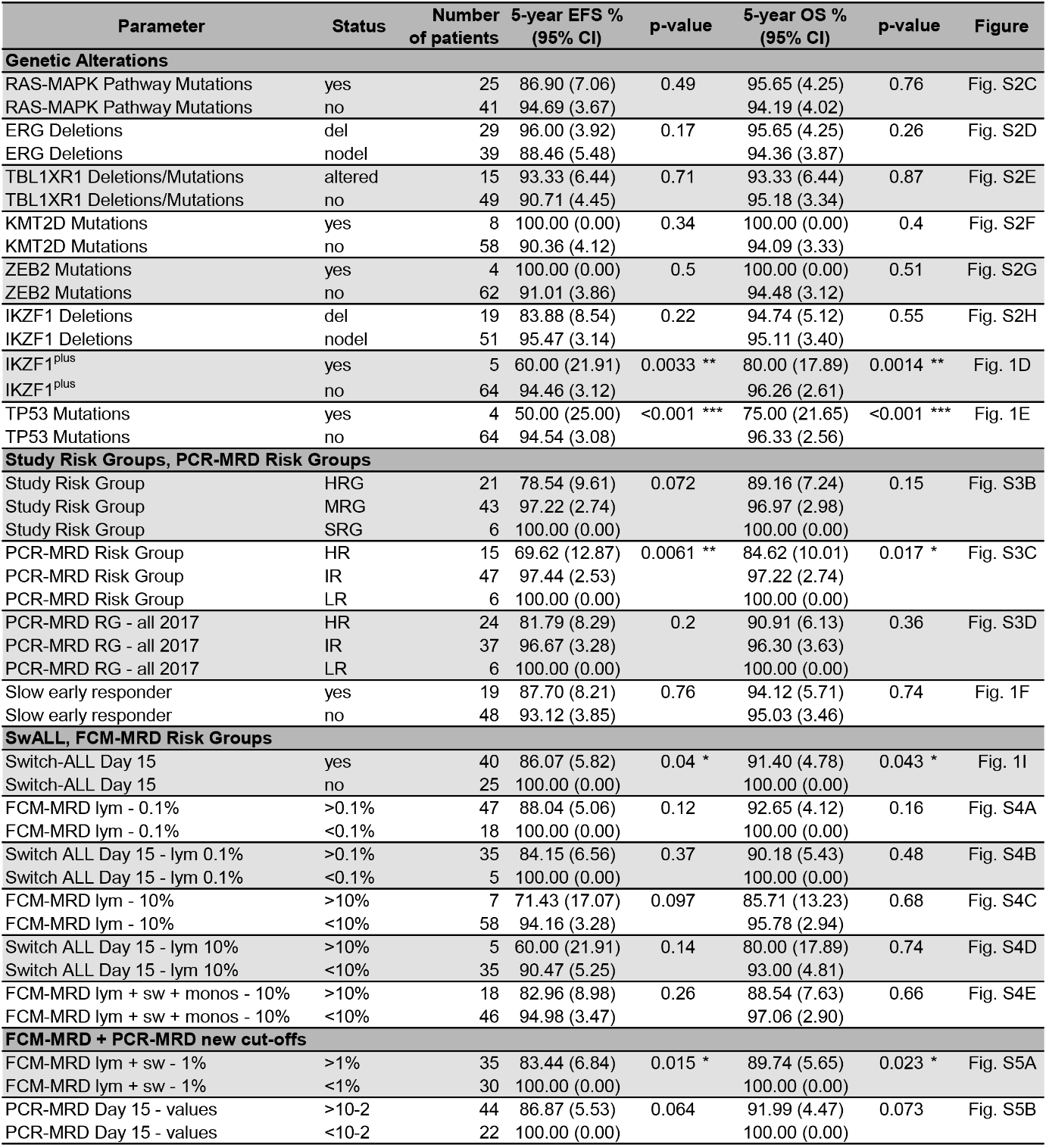


Supplementary Table S5**. Outcome data according to mutations, PCR-/FCM-MRD, and switch-ALL**. The 5-year event-free survival (EFS) and overall survival (OS) rates for various parameters of the Austrian DUX4-cohort including significance scores are summarized. del, deletion; nodel, no deletion; altered, mutation or deletion in *TBL1XR1*; SRG, standard risk group (RG); MRG, medium RG; HRG, high RG; FLR, HR, measurable residual disease (MRD) high risk; MR, MRD medium risk; LR, MRD low risk; SER, slow early response; lymphoblasts (lym); switch blasts (sw); monocytes (monos). Log-ranks test p‑values for EFS and OS. Stars indicate significance levels: * <0.05; **, <0.01; *** <0.001.


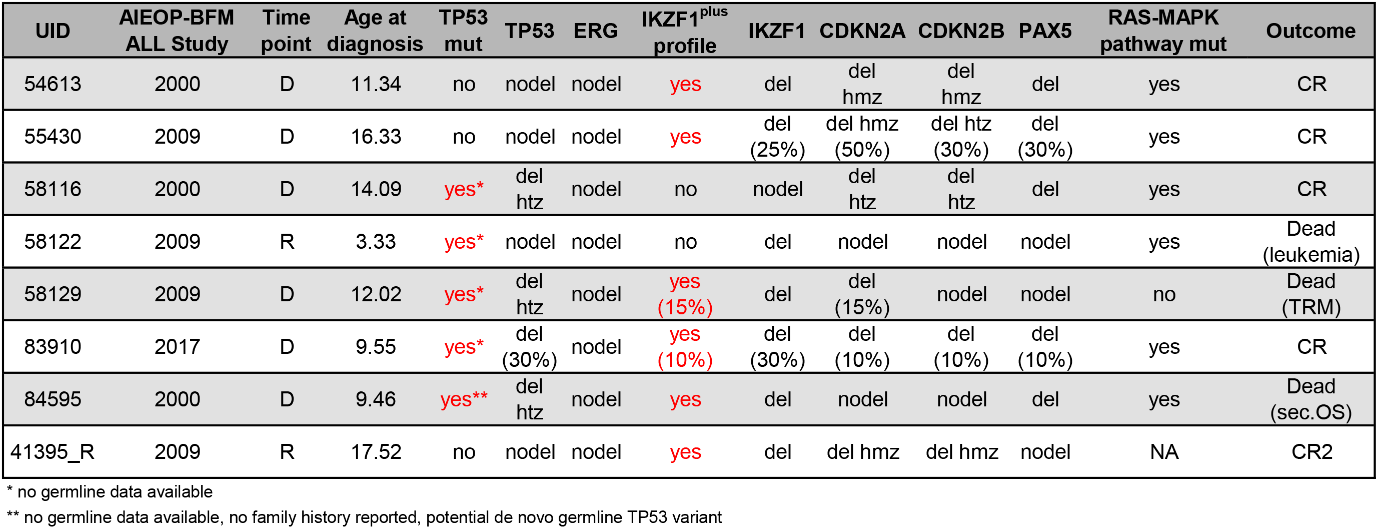


Supplementary Table S6**.** **Characteristics of *TP53*-mutated and/or IKZF1^plus^ DUX4 cases.** The most relevant copy number alterations defining the IKZF1^plus^ deletion profile are provided, including the percentage of deleted cells in cases with subclonal deletions detected by SNP array analysis. UID, unique patient identifier; R, relapse; Time point D, diagnosis; R, relapse; Age at diagnosis, years; del, deletion; nodel, no deletion; htz, heterozygous; hmz, homozygous; mut, mutation; CR, complete remission; CR2, second complete remission; TRM, treatment-related mortality; sec. OS, secondary osteosarcoma. NA, not available.





Supplementary Table S7. **Actual risk group (RG) and risk group according to AIEOP-BFM ALL 2017.** RG assignments showing the differences between the actual study RG and the RG assignment according to AIEOP-BFM ALL 2017 criteria. SRG, standard RG; MRG, medium RG; HRG, high RG; LR, measurable residual disease (MRD) measured by PCR; LR, MRD low risk; IR, MRD intermediate risk; HR, MRD high risk.


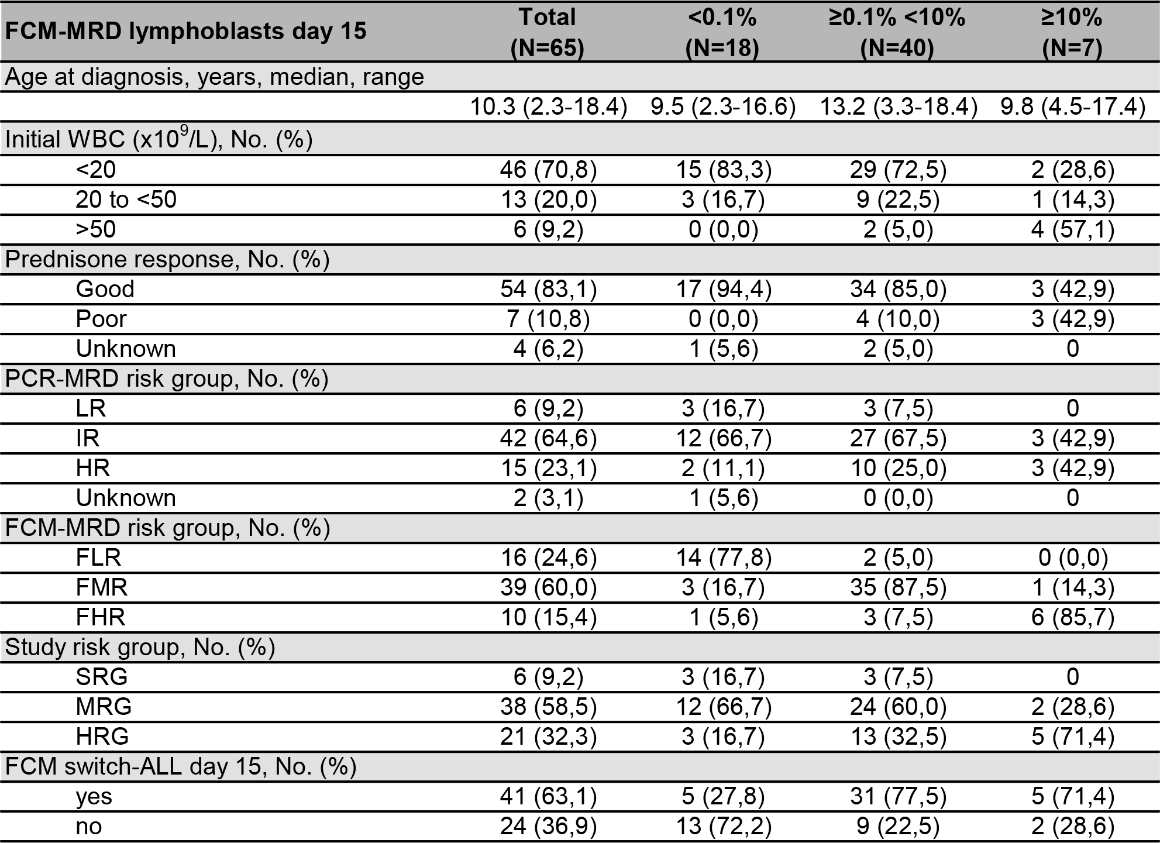


Supplementary Table S8**. Characteristics of DUX4 patients according to FCM-MRD lymphoblasts at day 15.** LR, low risk; IR, intermediate risk; HR, high risk; FLR, flow cytometry low risk; FMR, flow cytometry medium risk; FHR, flow cytometry high risk; SRG, standard risk group; MRG, medium risk group; HRG, high risk group. FCM, flow cytometry; MRD, measurable residual disease; WBC, white blood cell count.

# Supplementary Figures





Supplementary Figure S1**. Identification of DUX4 positive patients. ***From the initial starting cohort of 1237 patients tested positive for *ETV6::RUNX1*, *TCF3::PBX1*, *BCR::ABL1*, *TCF3::HLF*, *KMT2A::AFF1*, other *KMT2A* rearrangements, high hyperdiploidy (51-67 chromosomes, near haploidy (23-29 chromosomes), low hypodiploidy (33-39 chromosomes), and intrachromosomal amplification of chromosome 21 (iAMP21) were excluded. **Nine patients could not be fully analyzed due to the lack of material. Patients with *ERG* deletion by SNP array and *IGH::DUX4-*positive by genomic PCR (Lilljebjörn et al., manuscript in preparation) were classified as DUX4. ***Patients with rearrangements and/or mutations typical of other subtypes as determined by SNP array, FISH and/or RT-PCR or mutation screening for PAX5 P80R and/or IKZF1 N159Y were excluded. Genetically unclassified patients were analyzed according their immunophenotype determined by flow cytometry: patients positive for CD371 and positive for CD2 (n=1) were selected as DUX4 cases. In total, n=70 DUX4 (n=0 with DS, Down syndrome) were identified.


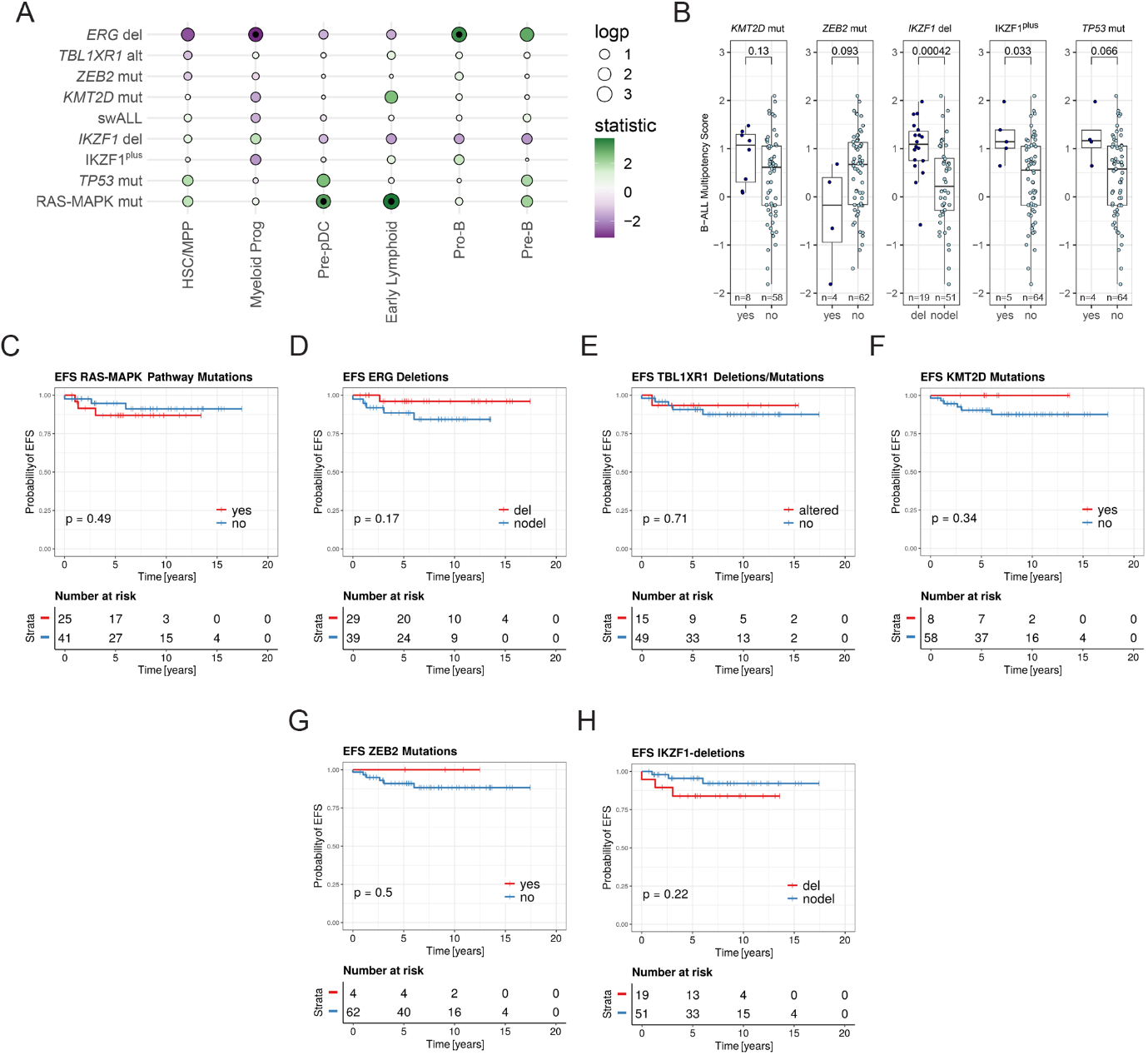


Supplementary Figure S2**. Developmental states and outcome according to mutations. (A)** Associations between alterations and inferred abundance of B-ALL developmental states by RNA-seq. The magnitude of each association, quantified as the –log10 (p value), is indicated by the size and color intensity of each dot. The direction of the association is indicated by the color, with higher abundance in green and lower abundance in purple. Associations with an FDR <0.05 are indicated by a black dot; del, deletion; alt, alteration (including mutations and/or deletions); mut, mutation; swALL, DUX4 with switch toward the monocytic lineage determined by flow cytometry (FCM) at day 15; HSC/MPP, hematopoietic stem cell/multipotent progenitor; prog, progenitor; pre-pDC, plasmacytoid pre-dendritic cells; early lymphoid, early lymphoid progenitor; Pro-B, pro-B cell; Pre-B, pre-B cell state. **(B)** B-ALL multipotency score for different alterations in DUX4 B-ALL; mut, mutation; del, deletion; alt, mutation and/or deletion; numbers of cases per subgroup are depicted at the bottom of the boxplots; Wilcoxon rank sum p-value. **(C-H)** Kaplan-Meier survival curves of event-free survival (EFS) based on **(C)** RAS-MAPK pathway mutation status; red, mutated; blue, no mutation. **(D)** *ERG* deletion status; red, *ERG* deletion; blue, no *ERG* deletion. **(E)** *TBL1XR1* alt, mutations and/or deletions in *TBL1XR1*; red, altered; blue, no alteration in *TBL1XR1*. **(F)** *IKZF1* deletion status; red, deletion; blue, no deletion. **(G)** *KMT2D* mutation status; red, mutated; blue, no mutation. **(H)** *ZEB2* mutation status; red, mutated; blue, no mutation. Log-ranks test p‑values for EFS.


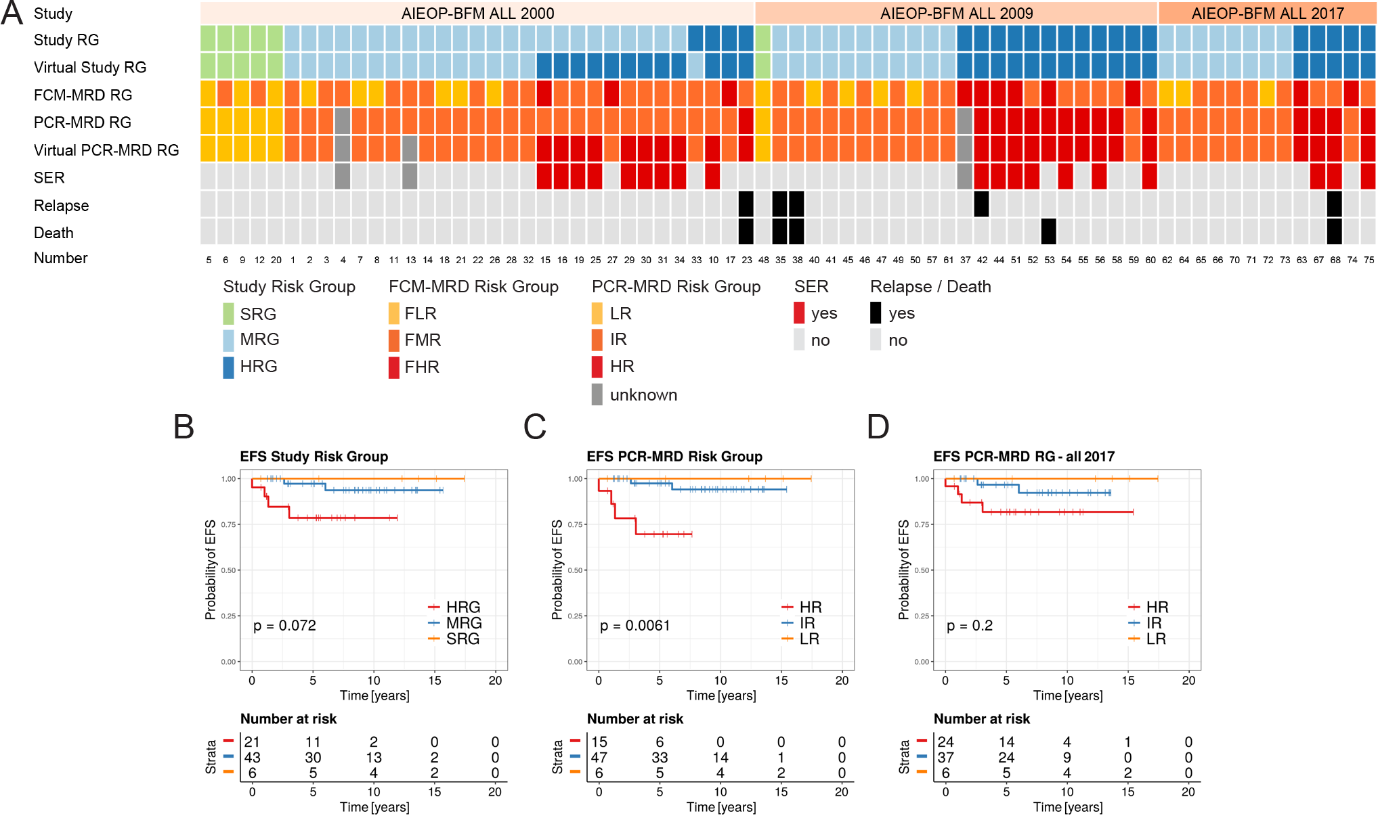


Supplementary Figure S3**. Overview actual and AIEOP-BFM ALL 2017 risk group (RG) assignment of DUX4 patients.** (**A**) Summary of the risk group assignment ordered by clinical trial showing the actual study risk group and the virtual risk group assignment according to AIEOP-BFM ALL 2017 criteria. SRG, standard RG; MRG, medium RG; HRG, high RG; FLR, flow cytometry (FCM) low risk; FMR, FCM medium risk; FHR, FCM high risk; LR, measurable residual disease (MRD) low risk; MR, MRD medium risk; HR, MRD high risk; SER, slow early response; depicted numbers correspond to patient numbers in Supplementary Table S1. Kaplan-Meier survival curves of event-free (EFS) based on (**B**) actual study risk groups; red, high RG, blue, medium RG, orange, standard RG; (**C**) actual PCR-MRD RG; red, high RG, blue, intermediate RG, orange, low RG; (**D**) PCR-MRD RG according to AIEOP-BFM ALL 2017 criteria; red, high RG, blue, intermediate RG, orange, low RG. Log-ranks test p-values for EFS.


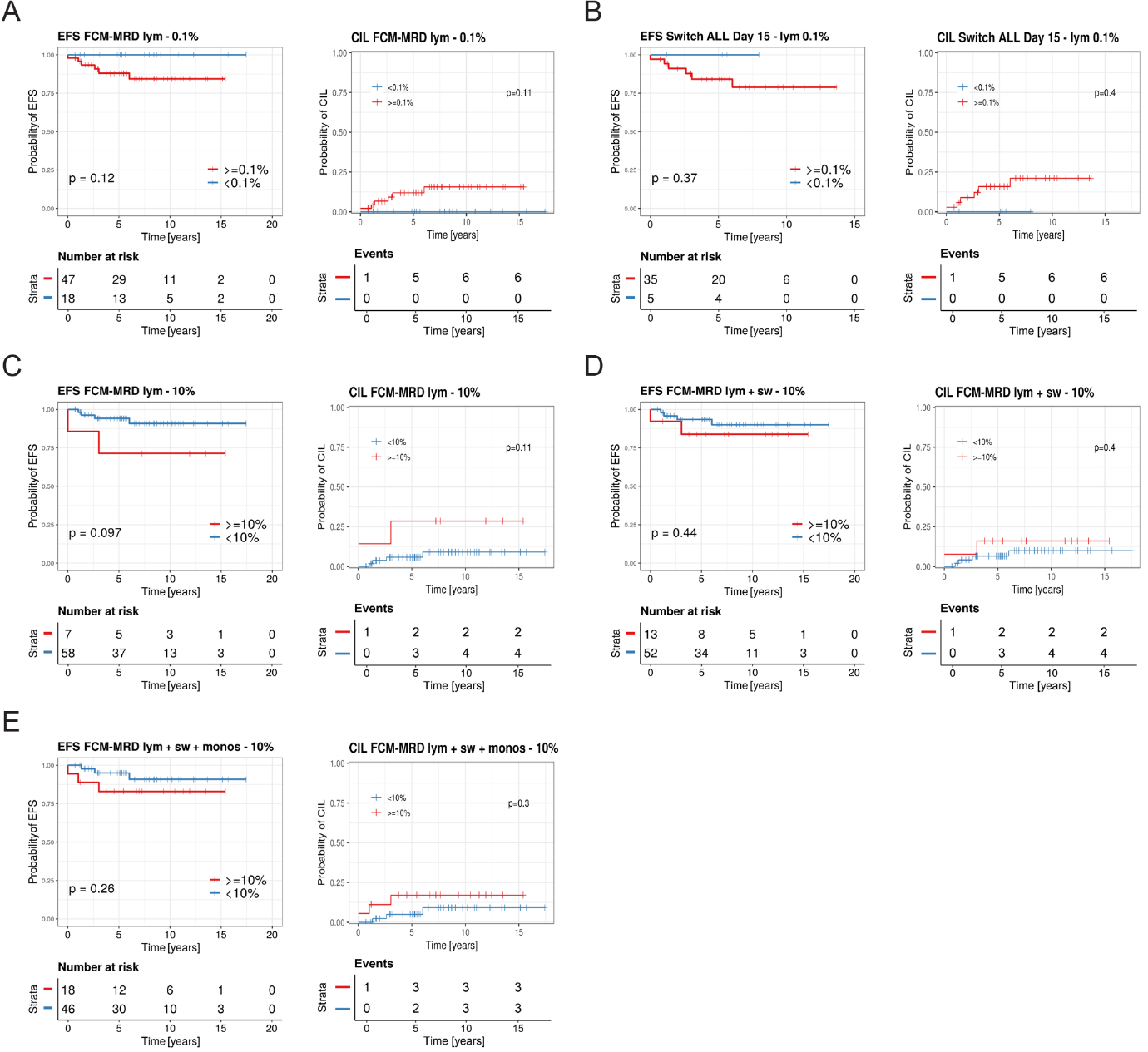


Supplementary Figure S4. **Impact of FCM-MRD stratification on outcome.** Kaplan-Meier survival curves of event-free survival (EFS) and cumulative incidence of leukemia-related events (CIL) based on re-gated FCM-MRD-values **(A)** including lymphoblasts (lym), blue <0.1%, red ≥0.1%; **(B)** including only lymphoblasts in DUX4 B-ALL with monocytic switch, blue <0.1%, red ≥0.1%; **(C)** including lymphoblasts, blue <10%, red ≥10%; **(D)** including lymphoblasts (lym) and switch blasts (sw), blue <10%, red ≥10%; **(E)** including lymphoblasts (lym), switch blasts (sw) and monocytes (monos), blue <10%, red ≥10%. Log-ranks test p‑values for EFS and Gray's test p-values for CIL. FCM, flow cytometry; MRD, measurable residual disease.


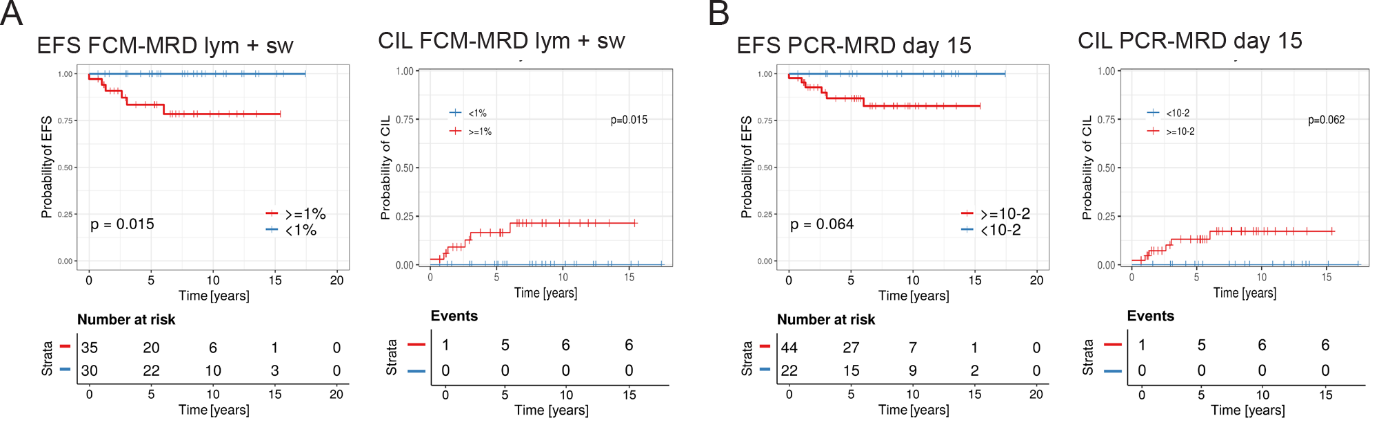


Supplementary Figure S5. **Impact of alternative FCM- and PCR-MRD-based cutoffs on outcome.** Kaplan-Meier survival curves of event-free survival (EFS) and cumulative incidence of leukemia-related events (CIL) based on **(A)** re-gated FCM-MRD-values including lymphoblasts (lym) and switch blasts (sw), blue <1%, red ≥1%; **(B)** PCR-MRD-values measured at day 15, blue <10^-2^, red ≥10^-2^. Log-ranks test p-values for EFS and Gray's test p‑values for CIL. FCM, flow cytometry; MRD, measurable residual disease.

# Supplementary Files

Supplementary Table S1. **Count table DUX4**

Raw count table of htseq-counts of DUX4 positive samples (66 diagnosis samples, 2 relapse samples); colnames fit unique patient identifier (UID) listed in Supplementary Table 1 and 2.

Supplementary Table S2. **Genetic characteristics and clinical parameter DUX4 patients (Diagnosis)**

Supplementary Table S3. **Genetic characteristics and clinical parameter DUX4 patients (Relapse)**

# Supplementary References

1. Busse TM, Roth JJ, Wilmoth D, Wainwright L, Tooke L, Biegel JA. Copy number alterations determined by single nucleotide polymorphism array testing in the clinical laboratory are indicative of gene fusions in pediatric cancer patients. Genes Chromosomes Cancer. 2017; 56:730-749.

2. Schieck M, Lentes J, Thomay K, Hofmann W, Behrens YL, Hagedorn M, et al. Implementation of RNA sequencing and array CGH in the diagnostic workflow of the AIEOP-BFM ALL 2017 trial on acute lymphoblastic leukemia. Ann Hematol. 2020; 99:809-818.

3. Abbasi MR, Nebral K, Haslinger S, Inthal A, Zeitlhofer P, Konig M, et al. Copy Number Changes and Allele Distribution Patterns of Chromosome 21 in B Cell Precursor Acute Lymphoblastic Leukemia. Cancers (Basel). 2021; 13.

4. Schwab CJ, Jones LR, Morrison H, Ryan SL, Yigittop H, Schouten JP, et al. Evaluation of multiplex ligation-dependent probe amplification as a method for the detection of copy number abnormalities in B-cell precursor acute lymphoblastic leukemia. Genes Chromosomes Cancer. 2010; 49:1104-1113.

5. Stanulla M, Dagdan E, Zaliova M, Moricke A, Palmi C, Cazzaniga G, et al. IKZF1(plus) Defines a New Minimal Residual Disease-Dependent Very-Poor Prognostic Profile in Pediatric B-Cell Precursor Acute Lymphoblastic Leukemia. J Clin Oncol. 2018; 36:1240-1249.

6. Schinnerl D, Mejstrikova E, Schumich A, Zaliova M, Fortschegger K, Nebral K, et al. CD371 cell surface expression: a unique feature of DUX4-rearranged acute lymphoblastic leukemia. Haematologica. 2019; 104:e352-e355.

7. Dobin A, Gingeras TR. Mapping RNA-seq Reads with STAR. Curr Protoc Bioinformatics. 2015; 51:11 14 11-19.

8. Wickham H. ggplot2: Elegant Graphics for Data Analysis: Springer-Verlag New York. 2016.; 2016.

9. Nicorici D, Satalan M, Edgren H, Kangaspeska S, Murumagi A, Kallioniemi O, et al. FusionCatcher – a tool for finding somatic fusion genes in paired-end RNA-sequencing data. bioRxiv. 2014:doi: 10.1101/011650.

10. Uhrig S, Ellermann J, Walther T, Burkhardt P, Frohlich M, Hutter B, et al. Accurate and efficient detection of gene fusions from RNA sequencing data. Genome Res. 2021; 31:448-460.

11. Tian L, Li Y, Edmonson MN, Zhou X, Newman S, McLeod C, et al. CICERO: a versatile method for detecting complex and diverse driver fusions using cancer RNA sequencing data. Genome Biol. 2020; 21:126.

12. Rusch M, Nakitandwe J, Shurtleff S, Newman S, Zhang Z, Edmonson MN, et al. Clinical cancer genomic profiling by three-platform sequencing of whole genome, whole exome and transcriptome. Nat Commun. 2018; 9:3962.

13. Newman S, Nakitandwe J, Kesserwan CA, Azzato EM, Wheeler DA, Rusch M, et al. Genomes for Kids: The Scope of Pathogenic Mutations in Pediatric Cancer Revealed by Comprehensive DNA and RNA Sequencing. Cancer Discov. 2021.

14. Mullighan CG, Zhang J, Kasper LH, Lerach S, Payne-Turner D, Phillips LA, et al. CREBBP mutations in relapsed acute lymphoblastic leukaemia. Nature. 2011; 471:235-239.

15. Holmfeldt L, Wei L, Diaz-Flores E, Walsh M, Zhang J, Ding L, et al. The genomic landscape of hypodiploid acute lymphoblastic leukemia. Nat Genet. 2013; 45:242-252.

16. Shah S, Schrader KA, Waanders E, Timms AE, Vijai J, Miething C, et al. A recurrent germline PAX5 mutation confers susceptibility to pre-B cell acute lymphoblastic leukemia. Nat Genet. 2013; 45:1226-1231.

17. Roberts KG, Li Y, Payne-Turner D, Harvey RC, Yang YL, Pei D, et al. Targetable kinase-activating lesions in Ph-like acute lymphoblastic leukemia. N Engl J Med. 2014; 371:1005-1015.

18. Andersson AK, Ma J, Wang J, Chen X, Gedman AL, Dang J, et al. The landscape of somatic mutations in infant MLL-rearranged acute lymphoblastic leukemias. Nat Genet. 2015; 47:330-337.

19. Zhang J, McCastlain K, Yoshihara H, Xu B, Chang Y, Churchman ML, et al. Deregulation of DUX4 and ERG in acute lymphoblastic leukemia. Nat Genet. 2016; 48:1481-1489.

20. Gu Z, Churchman ML, Roberts KG, Moore I, Zhou X, Nakitandwe J, et al. PAX5-driven subtypes of B-progenitor acute lymphoblastic leukemia. Nat Genet. 2019; 51:296-307.

21. Waanders E, Gu Z, Dobson SM, Antic Z, Crawford JC, Ma X, et al. Mutational landscape and patterns of clonal evolution in relapsed pediatric acute lymphoblastic leukemia. Blood Cancer Discov. 2020; 1:96-111.

22. McLeod C, Gout AM, Zhou X, Thrasher A, Rahbarinia D, Brady SW, et al. St. Jude Cloud: A Pediatric Cancer Genomic Data-Sharing Ecosystem. Cancer Discov. 2021; 11:1082-1099.

23. Love MI, Huber W, Anders S. Moderated estimation of fold change and dispersion for RNA-seq data with DESeq2. Genome Biol. 2014; 15:550.

24. Harvey RC, Mullighan CG, Wang X, Dobbin KK, Davidson GS, Bedrick EJ, et al. Identification of novel cluster groups in pediatric high-risk B-precursor acute lymphoblastic leukemia with gene expression profiling: correlation with genome-wide DNA copy number alterations, clinical characteristics, and outcome. Blood. 2010; 116:4874-4884.

25. Yeoh EJ, Ross ME, Shurtleff SA, Williams WK, Patel D, Mahfouz R, et al. Classification, subtype discovery, and prediction of outcome in pediatric acute lymphoblastic leukemia by gene expression profiling. Cancer Cell. 2002; 1:133-143.

26. Beder T, Hansen BT, Hartmann AM, Zimmermann J, Amelunxen E, Wolgast N, et al. The Gene Expression Classifier ALLCatchR Identifies B-cell Precursor ALL Subtypes and Underlying Developmental Trajectories Across Age. Hemasphere. 2023; 7:e939.

27. Schmidt B, Brown LM, Ryland GL, Lonsdale A, Kosasih HJ, Ludlow LE, et al. ALLSorts: an RNA-Seq subtype classifier for B-cell acute lymphoblastic leukemia. Blood Adv. 2022; 6:4093-4097.

28. Iacobucci I, Zeng AGX, Gao Q, Garcia-Prat L, Baviskar P, Shah S, et al. Single Cell Dissection of Developmental Origins and Transcriptional Heterogeneity in B-Cell Acute Lymphoblastic Leukemia. bioRxiv. 2023.

29. DePristo MA, Banks E, Poplin R, Garimella KV, Maguire JR, Hartl C, et al. A framework for variation discovery and genotyping using next-generation DNA sequencing data. Nat Genet. 2011; 43:491-498.

30. Kircher M, Witten DM, Jain P, O'Roak BJ, Cooper GM, Shendure J. A general framework for estimating the relative pathogenicity of human genetic variants. Nat Genet. 2014; 46:310-315.

31. McKenna A, Hanna M, Banks E, Sivachenko A, Cibulskis K, Kernytsky A, et al. The Genome Analysis Toolkit: a MapReduce framework for analyzing next-generation DNA sequencing data. Genome Res. 2010; 20:1297-1303.

32. McLaren W, Gil L, Hunt SE, Riat HS, Ritchie GR, Thormann A, et al. The Ensembl Variant Effect Predictor. Genome Biol. 2016; 17:122.

33. Van der Auwera GA, O’Connor BD. Genomics in the Cloud: Using Docker, GATK, and WDL in Terra (1st Edition). O'Reilly Media. 2020.

34. Hrusak O, Basso G, Ratei R, Gaipa G, Luria D, Mejstrikova E, et al. AIEOP-BFM Flow Network. Flow diagnostics essential code: a simple and brief format for the summary of leukemia phenotyping. Cytometry B Clin Cytom. 2014; 86:288-291.

35. Dworzak MN, Buldini B, Gaipa G, Ratei R, Hrusak O, Luria D, et al. AIEOP-BFM consensus guidelines 2016 for flow cytometric immunophenotyping of Pediatric acute lymphoblastic leukemia. Cytometry B Clin Cytom. 2018; 94:82-93.

36. Buldini B, Varotto E, Maurer-Granofszky M, Gaipa G, Schumich A, Bruggemann M, et al. CD371+ pediatric B-cell acute lymphoblastic leukemia: propensity to lineage switch and slow early response to treatment. Blood. 2024.

37. Maurer-Granofszky M, Schumich A, Buldini B, Gaipa G, Kappelmayer J, Mejstrikova E, et al. An Extensive Quality Control and Quality Assurance (QC/QA) Program Significantly Improves Inter-Laboratory Concordance Rates of Flow-Cytometric Minimal Residual Disease Assessment in Acute Lymphoblastic Leukemia: An I-BFM-FLOW-Network Report. Cancers (Basel). 2021; 13.

38. Schumich A, Maurer-Granofszky M, Attarbaschi A, Potschger U, Buldini B, Gaipa G, et al. Flow-cytometric minimal residual disease monitoring in blood predicts relapse risk in pediatric B-cell precursor acute lymphoblastic leukemia in trial AIEOP-BFM-ALL 2000. Pediatr Blood Cancer. 2019; 66:e27590.

39. Dworzak MN, Gaipa G, Ratei R, Veltroni M, Schumich A, Maglia O, et al. Standardization of flow cytometric minimal residual disease evaluation in acute lymphoblastic leukemia: Multicentric assessment is feasible. Cytometry B Clin Cytom. 2008; 74:331-340.

40. Bouriche L, Bernot D, Nivaggioni V, Arnoux I, Loosveld M. Detection of Minimal Residual Disease in B Cell Acute Lymphoblastic Leukemia Using an Eight-Color Tube with Dried Antibody Reagents. Cytometry B Clin Cytom. 2019; 96:158-163.

41. Cazzaniga G, Songia S, Biondi A, Euro MRDWG. PCR Technology to Identify Minimal Residual Disease. Methods Mol Biol. 2021; 2185:77-94.

42. van der Velden VH, Cazzaniga G, Schrauder A, Hancock J, Bader P, Panzer-Grumayer ER, et al. Analysis of minimal residual disease by Ig/TCR gene rearrangements: guidelines for interpretation of real-time quantitative PCR data. Leukemia. 2007; 21:604-611.

43. Conter V, Bartram CR, Valsecchi MG, Schrauder A, Panzer-Grumayer R, Moricke A, et al. Molecular response to treatment redefines all prognostic factors in children and adolescents with B-cell precursor acute lymphoblastic leukemia: results in 3184 patients of the AIEOP-BFM ALL 2000 study. Blood. 2010; 115:3206-3214.

44. Kaplan EL, Meier P. Nonparametric estimation from incomplete observations. J Amer Statist Assoc. 1958; 53:457-481.

45. Kalbfleisch JD, Prentice RL. The Statistical Analysis of Failure Time Data. New York: John Wiley & Sons; 1980.

46. Cox DR. Regression Models and Life-Tables. Journal of the Royal Statistical Society Series B (Methodological). 1972; 34:187-220.

47. Lilljebjorn H, Henningsson R, Hyrenius-Wittsten A, Olsson L, Orsmark-Pietras C, von Palffy S, et al. Identification of ETV6-RUNX1-like and DUX4-rearranged subtypes in paediatric B-cell precursor acute lymphoblastic leukaemia. Nat Commun. 2016; 7:11790.

48. Yasuda T, Tsuzuki S, Kawazu M, Hayakawa F, Kojima S, Ueno T, et al. Recurrent DUX4 fusions in B cell acute lymphoblastic leukemia of adolescents and young adults. Nat Genet. 2016; 48:569-574.
